# Usefulness and safety of a dedicated team to prone patients with severe ARDS due to COVID-19

**DOI:** 10.1186/s13054-020-03128-6

**Published:** 2020-08-18

**Authors:** Antoine Kimmoun, Bruno Levy, Bruno Chenuel, The DV-Team group, S. Barde, A. Didelot, B. Chenuel, P. Zieminski, M. Lorcin, C. Schweitzer, R. Karunna, V. Moulin, M. Huet, F. Lacour, P. Rodriguez, D. Grandmougin, Y. Liu, P. A. Metzdorf, M. Costa, T. Fouquet, A. Germain, H. Chanty, J. Levy, A. Didier, J. Lawton, C. Parietti-Winkler, A. Manuguerra, C. Mazeaud, C. Gaulier, C. Rumeau, L. Bourson, M. Cholley-Roulleau, A. Ionescu, A. Gatin, M. Perez, A. Schwanké, G. Lauria, L. Freysz, T. Cuinet, J. Chauvelot, C. Mottola, T. Toussaint, L. Dechambenoit, F. Lagrange, C. Mathieu, C. Clément, H. Benamron, L. Dubouis, H. Kremer, L. Cabanel, M. Falcetta, V. Gorzkowski, P. Campoli, J. Cavailhes, J. Zavoli, C. Nominé-Criqui, J. Felloni, V. Cloché-Fouquet, F. G. Midon, G. Vaz, D. Valentin, V. Perkovic, A. Courandon, Y. Briot, A. Epin, K. Dreller, L. Florion, C. Larose, M. Barron, C. Sadoul, M. Gimbert, M. Fernandez, T. Thomas, P. Bichet, N. Petkunaite, S. Brahami, D. Nguyen, G. Vaz, A. Schaefer, C. Fabbri, C. Ferri, A. Gegout, A. Poncy, R. Delaplace, M. Ammisaid, J. Rebois, B. Vendeville, C. Dubroux, R. Raynaud, S. Moog, C. Cottez, L. Woirhaye, J. Menet, A. C. Madkaud, L. Naisseline, C. Mathieu, T. Raze, F. Violon, M. Meiers, D. Albanesi, O. Durand, L. Textoris, T. Dubost

**Affiliations:** 1 CHRU-Nancy, INSERM U1116, Médecine Intensive et Réanimation Brabois, Université de Lorraine, F-54000 Nancy, France; 2 CHRU-Nancy, Centre Universitaire de Médecine du Sport et Activité Physique Adaptée, Explorations Fonctionnelles Respiratoires, EA DevAH, Département de Physiologie, Université de Lorraine, F-54000 Nancy, France; 3 CHRU-Nancy, Centre Universitaire de Médecine du Sport et Activité Physique Adaptée, Université de Lorraine, F-54000 Nancy, France; 4 CHRU-Nancy, Médecine Vasculaire, Université de Lorraine, F-54000 Nancy, France; 5 Faculté de Médecine de Nancy, CHRU-Nancy, Université de Lorraine, F-54000 Nancy, France; 6 CHRU-Nancy, ORL et Chirurgie Cervico-Faciale, Université de Lorraine, F-54000 Nancy, France; 7 CHRU-Nancy, Chirurgie Cardio-Vasculaire et Transplantations, Université de Lorraine, F-54000 Nancy, France; 8 CHRU-Nancy, Cardiologie Médicale, Université de Lorraine, F-54000 Nancy, France; 9 CHRU-Nancy, Unité Médicochirurgicale de Chirurgie Viscérale et Cancérologie, Université de Lorraine, F-54000 Nancy, France; 10 CHRU-Nancy, Chirurgie Digestive et Générale, Université de Lorraine, F-54000 Nancy, France; 11 CHRU-Nancy, Biologie Médicale, Université de Lorraine, F-54000 Nancy, France; 12 CHRU-Nancy, Hépato-Gastro-Entérologie, Université de Lorraine, F-54000 Nancy, France; 13 CHRU-Nancy, Urologie, Université de Lorraine, F-54000 Nancy, France; 14 CHRU-Nancy, Chirurgie de la main, Université de Lorraine, F-54000 Nancy, France; 15 CHRU-Nancy, Pédiatrie, Université de Lorraine, F-54000 Nancy, France; 16 CHRU-Nancy, Chirurgie Orthopédique Infantile, Université de Lorraine, F-54000 Nancy, France; 17 CHRU-Nancy, Néphrologie, Université de Lorraine, F-54000 Nancy, France; 18 CHRU-Nancy, Anatomopathologie, Université de Lorraine, F-54000 Nancy, France; 19 CHRU-Nancy, Chirurgie Orthopédique, Université de Lorraine, F-54000 Nancy, France; 20 CHRU-Nancy, Gynécologie Obstétrique, Université de Lorraine, F-54000 Nancy, France; 21 CHRU-Nancy, Département de Médecine Générale, Université de Lorraine, F-54000 Nancy, France; 22 CHRU-Nancy, Rééducation Fonctionnelle, Université de Lorraine, F-54000 Nancy, France; 23 CHRU-Nancy, Ophtalmologie, Université de Lorraine, F-54000 Nancy, France; 24 CHRU-Nancy, Nutrition, Université de Lorraine, F-54000 Nancy, France; 25 CHRU-Nancy, Chirurgie Maxillo-Faciale et Stomatologique, Université de Lorraine, F-54000 Nancy, France; 26 CHRU-Nancy, Chirurgie Vasculaire et Endoluminale, Université de Lorraine, F-54000 Nancy, France; 27 CHRU-Nancy, Consultations Pathologies Professionnelles, Université de Lorraine, F-54000 Nancy, France; 28 CHRU-Nancy, Chirurgie Infantile Viscérale, Université de Lorraine, F-54000 Nancy, France; 29 CHRU-Nancy, Médecine Nucléaire, Université de Lorraine, F-54000 Nancy, France; 30 CHRU-Nancy, INSERM U1116, Médecine Intensive et Réanimation Brabois, Université de Lorraine, F-54000 Nancy, France

To the Editor:

Prone position ventilation has been shown to improve oxygenation and survival in patients with severe acute respiratory distress syndrome (ARDS) [1]. Facing the coronavirus disease 2019 (COVID-19) pandemic, prone positioning (PP) is of crucial importance to treat severe ARDS patients [2]. Nevertheless, the high number of ICU admissions quickly overwhelmed the ability of the daily ICU team to place patients in PP, a complex and time-consuming maneuver. Thus, we created a dedicated medical team with reassigned volunteers to cope with the large number of patients requiring PP.

PP Team consisted of five volunteers: a senior medical non-intensivist physician placed at the patient’s head to secure the endotracheal tube and four medical fellows or medical students placed at each side of the bed. For patients treated with VV-ECMO, a supplementary physician was added to secure the lines. Since PP is a complex procedure and has many potential adverse events requiring adequate and well-trained staff, volunteers received previously a theoretical training and a hands-on ad hoc training session. PP teams followed the guidelines for PP placement [1].

This retrospective observational study was performed in our extended ICU (from 22 to 46 beds), from the first day of deployment of PPT (March 23 to April 23, 2020).

The main characteristics and outcomes of prone positioned patients (*n* = 63) are presented in Table 1. A total of 367 placements in a prone or supine position were performed during the 1-month study period (Table 2).

**Table 1:** Initial characteristics and outcome of the prone positioned ARDS COVID-19 population (data are expressed as median (interquartile range, IQR) or number (%) as appropriate. After visual assumption of normality, Wilcoxon rank tests were applied for continuous variables. For categorical variables, Fisher’s exact or chi^2^ tests were applied as appropriate)

Variables	***n***, total available	Total, ***n***(%) or median (IQR) (***n*** = 63)	***n***, available for ICU survivors (***n*** = 46) *	ICU survivors, ***n***(%) or median (IQR) *	***n***, available for ICU non-survivors (***n*** = 16)*	ICU non-survivors, ***n***(%) or median (IQR) *	***p***
**Demographic data**							
Female ratio	63	15 (24%)	46	10 (22%)	16	5 (31%)	0.50
Age (years)	63	64 (56–70)	46	62 (54–69)	16	67 (64–74)	0.045
Weight (kg)	61	89 (75–103)	45	90 (80–103)	15	89 (73–106)	0.68
Body mass index (kg/m^2^)	61	30 (25–36)	45	30 (26–35)	15	29 (25–46)	0.87
SAPS II	63	42 (31–57)	46	37 (27–57)	16	46 (42–59)	0.030
**Medical history**							
Diabetes mellitus	63	17 (27%)	46	11 (24%)	16	5 (31%)	0.74
Hypertension	63	30 (48%)	46	19 (41%)	16	10 (62%)	0.14
Chronic respiratory disease	63	16 (25%)	46	13 (28%)	16	3 (19%)	0.53
Chronic immunosuppression†	63	5 (8%)	46	2 (4%)	16	3 (19%)	0.10
Chronic Cardiovascular disease	63	16 (25%)	46	12 (26%)	16	3 (19%)	0.74
Chronic kidney disease	63	3 (5%)	46	2 (4%)	16	1 (6%)	1.00
**Respiratory parameters**							
Static compliance (ml/cmH_2_O) before first prone positioning	46	33 (23–42)	35	35 (27–44)	11	22 (18–36)	0.036
PaO_2_/F_I_O_2_ ratio before first prone positioning	63	92 (70–117)	46	96 (70–120)	16	86 (64–111)	0.54
Number of prone positioning per patient	63	3 (2–6)	46	3 (2–6)	16	4 (3–8)	0.19
**Events in ICU**							
Vasopressors administered	63	38 (60%)	46	23 (50%)	16	14 (88%)	0.008
VV-ECMO	63	14 (22%)	46	11 (24%)	16	3 (19%)	1.00
Renal replacement therapy	63	8 (13%)	46	4 (9%)	16	4 (25%)	0.19
ICU length of stay (days)	62	19 (14–31)	46	20 (15–32)	16	16 (12–28)	0.24

^*^1 patient still in ICU

^†^Representing active cancer medical history or chronic immunosuppressor therapies

**Table 2:** Prone and supine placements and reported adverse events during the procedure on the study period. (data are expressed as mean ± standard deviation or number (percentage)). The 367 placements represent the placement in prone or supine positions

	Prone/Supine positioning placements, ***n*** (%) or mean ± SD (***n*** = 367)
**Number of placements performed**	
Daily	11.5 ± 3.4
First 2-day period	7 ± 1.4
Acme 2-day period	20 ± 4.2
Last 2-day period	5 ± 0
Under VV-ECMO	124 (34%)
**Adverse events recorded during placements**	
**Major**	
Cardiac arrest	0
Unscheduled extubation	0
Severe desaturation (SpO_2_ < 85%)*	5 (1%)
**Minor**	
Accidental device removing or disconnection†	6 (2%)

*Needing medical intervention

†Minor: one epistaxis following accidental removing of naso-gastric tube, four incidental disconnections of ventilator lines, one incidental removing of central venous catheter

This specific medical team of trained non-intensivist volunteers was able to manage this delicate PP task without any major adverse events such as cardiac arrest or unscheduled extubation when compared to the relatively high incidence (respectively 6.8 and 13.3%) observed in Guérin et al. study [1]. Our studied population was comparable to already published series of severe ARDS, and we found a similar mortality (26%) despite a lower initial P/F ratio and COVID-19 association [1]. Interestingly, we recorded a greater survival rate than reported by Richardson et al. in the New York area at their edge of COVID-19 pandemic, but they did not detail the use of PP [3].

This innovative management allowed three major benefits: (i) critical relief of permanent intensive care team’s workload; (ii) reduction of the nurse-to-patient ratio, permitting also the reassignment of critical care nurses to newly created ICUs; and (iii) devoid of any self-censorship for fear of overwork and burn-out, intensivist physicians were able to strictly follow PP guideline recommendations, ensuring the best standard of care for ARDS patients.

Since the pathophysiology is poorly understood [4, 5], the specific role of PP among the optimal management for COVID-19 patients with ARDS, in order to reduce mortality needs to be addressed.

## Data Availability

All data generated or analyzed during this study are included in this published article. The data used to support the findings of this study are available from the corresponding author upon request.

## References

[1] Guérin C, Reignier J, Richard JC, Beuret P, Gacouin A, Boulain T (2013). Prone positioning in severe acute respiratory distress syndrome. N Engl J Med.

[2] Carsetti A, Damia Paciarini A, Marini B, et al. Prolonged prone position ventilation for SARS-CoV-2 patients is feasible and effective. Crit Care. 2020;24:225. 10.1186/s13054-020-02956-w PMC7226707 32414420

[3] Richardson S, Hirsch JS, Narasimhan M, Crawford JM, McGinn T, Davidson KW, the Northwell COVID-19 Research Consortium. Presenting characteristics, comorbidities, and outcomes among 5700 patients hospitalized with COVID-19 in the New York city area. JAMA. 2020. 10.1001/jama.2020.6775. 10.1001/jama.2020.6775 PMC7177629 32320003

[4] Gattinoni L, Chiumello D, Caironi P, Busana M, Romitti F, Brazzi L, et al. COVID-19 pneumonia: different respiratory treatments for different phenotypes? Intensive Care Med. 2020;46:1099–1102. 10.1007/s00134-020-06033-2. 10.1007/s00134-020-06033-2 PMC7154064 32291463

[5] Gattinoni L, Chiumello D, Rossi S. COVID-19 pneumonia: ARDS or not? Crit Care. 2020;24:154. 10.1186/s13054-020-02880-z. 10.1186/s13054-020-02880-z PMC7160817 32299472

